# Clinical features and outcomes of influenza and RSV coinfections: a report from Canadian immunization research network serious outcomes surveillance network

**DOI:** 10.1186/s12879-024-09033-5

**Published:** 2024-01-30

**Authors:** Henrique Pott, Jason J. LeBlanc, May S. ElSherif, Todd F. Hatchette, Shelly A. McNeil, Melissa K. Andrew, on behalf of the Serious Outcomes Surveillance (SOS) Network of the Canadian Immunization Research Network (CIRN)

**Affiliations:** 1 Canadian Center for Vaccinology, Dalhousie University, Halifax, Canada; 2 Department of Medicine, Universidade Federal de São Carlos, Rod. Washington Luis, km 235. São Carlos, São Carlos, 13656-905 Brazil; 3 Department of Pathology, Dalhousie University, Halifax, Canada; 4 Department of Medicine (Infectious Diseases), Dalhousie University, Halifax, Canada; 5 Department of Medicine (Geriatrics), Dalhousie University, Halifax, Canada; 6 Division of Microbiology, Department of Pathology and Laboratory Medicine, Nova Scotia Health, Halifax, Canada; 7 Department of Microbiology and Immunology, Dalhousie University, Halifax, Canada

**Keywords:** Influenza, Respiratory syncytial virus, Older adults, Outcomes

## Abstract

**Background:**

Influenza and RSV coinfections are not commonly seen but are concerning as they can lead to serious illness and adverse clinical outcomes among vulnerable populations. Here we describe the clinical features and outcomes of influenza and RSV coinfections in hospitalized adults.

**Methods:**

A cohort study was performed with pooled active surveillance in hospitalized adults ≥ 50 years from the Serious Outcomes Surveillance Network of the Canadian Immunization Research Network (CIRN SOS) during the 2012/13, 2013/14, and 2014/15 influenza seasons. Descriptive statistics summarized the characteristics of influenza/RSV coinfections. Kaplan-Meier estimated the probability of survival over the first 30 days of hospitalization.

**Results:**

Over three influenza seasons, we identified 33 cases of RSV and influenza coinfection, accounting for 2.39 cases per 1,000 hospitalizations of patients with acute respiratory illnesses. Adults aged 50 + years commonly reported cough (81.8%), shortness of breath (66.7%), sputum production (45.5%), weakness (33.3%), fever (27.3%), and nasal congestion (24.2%) as constitutional and lower respiratory tract infection symptoms. The mortality rate was substantial (12.1%), and age, comorbidity burden, and frailty were associated with a higher risk for adverse clinical outcomes.

**Conclusions:**

Older adults are at higher risk for complications from influenza and RSV coinfections, especially those over 65 with a high comorbidity burden and frailty.

**Supplementary Information:**

The online version contains supplementary material available at 10.1186/s12879-024-09033-5.

## Background

Respiratory syncytial virus (RSV) and influenza are respiratory viral infections that can cause serious illness in vulnerable individuals, especially those with a weakened immune system or other underlying comorbidities [1]. The seasonality of RSV varies somewhat between years and geographical locations. Like the influenza virus, epidemics of RSV are generally observed in temperate climates during the winter months; however, this is less predictable in tropical and subtropical regions [2]. Additionally, the pattern of occurrence of RSV epidemics can vary significantly between countries and regions within a country. Influenza virus typically peaks between late fall and early spring, and while there is overlap between influenza and RSV seasonality, there can be some temporal variations in the distribution of cases annually [3]. Coinfection with respiratory viruses, including RSV and influenza, is possible, as both are spread seasonally via contact with infected individuals or objects contaminated with viral particles [4, 5]. Infection with either virus is a serious concern as it can lead to severe illness and adverse clinical outcomes, particularly among older adults with comorbidities [6].

Both disease severity and the risk of hospitalization after infection increase with age [1]. Thus, the epidemiology of RSV and influenza in older adults is essential when considering prevention measures [7, 8]. Vaccination and infection control measures are the main preventive strategies for reducing RSV and influenza coinfection risk. In May 2023, the US Food and Drug Administration approved the first two vaccines to prevent RSV in adults aged 60 and over [9]. Meanwhile, vaccination against influenza is highly recommended to prevent infection, reduce its severity if contracted, and help prevent its transmission to others [10]. Management of RSV and influenza coinfection involves supportive care and antiviral medications, but clinical profiles of patients with coinfection and their outcomes are not well understood [10].

This report describes the demographic and clinical characteristics, disease presentation, and outcomes of older adults hospitalized with a laboratory-confirmed RSV and influenza coinfection. Specifically, our objectives were to determine this population’s clinical features and presenting characteristics and to investigate the clinical outcomes observed during the hospital stay of these patients.

## Methods

### Study design and participants

This study used pooled data from the Serious Outcomes Surveillance (SOS) Network of the Canadian Immunization Research Network (CIRN). As presented in detail elsewhere, the SOS network has conducted active inpatient surveillance with a focus on influenza in Canadian hospitals since 2009 [11]. The present study used data from influenza seasons in which respiratory virus tests were performed on a multiplex platform [12, 13]: 2012/2013, 2013/2014, and 2014/2015 (pooled sample size, *N* = 13,797). In these seasons, active surveillance was conducted in five Canadian provinces (British Columbia, Ontario, Quebec, New Brunswick, and Nova Scotia). This secondary analysis of the CIRN SOS Network database used data from hospitalized adults aged 50 + with broadly defined acute respiratory illness. The eligibility criteria were (1) available data on multiplex respiratory virus testing, (2) sociodemographic and clinical features/outcomes, and (3) a positive result on multiplex respiratory virus testing for both RSV and influenza. There were no exclusion criteria.

Participants provided informed consent for data, sample collection, and medical record screening per the local Research Ethics Boards’ requirements. The Research Ethics Boards approved the protocol of participating institutions (ClinicalTrials.gov Identifier: NCT01517191).

### Variables

CIRN SOS Network follows a standardized protocol for data collection. Demographic data included sex, age, and the person’s residential location before hospital admission. Health-related data included: smoking status, signs and symptoms present at hospital admission (feverishness, nasal congestion, headache, abdominal pain, malaise, cough, diarrhea, weakness, shortness of breath, vomiting, dizziness, sore throat, nausea, muscle aches, arthralgia, prostration, seizures, myalgia, sneezing, conjunctivitis, sputum production, chest pain, encephalitis, nose bleed, altered consciousness, chills, and anorexia), a requirement for assistance with tasks of everyday life and the types of help needed, as well as the need for extra support with activities of daily living. Influenza vaccination status was classified as “current season vaccination” for those who received a current season influenza vaccine more than 14 days before symptom onset, “vaccination in prior seasons only” for those who received a flu vaccine the flu in previous seasons but not the current one, and “never vaccinated” otherwise.

### Comorbidity burden and frailty assessment

We assessed the burden of comorbidities using Quan’s Updated Charlson Comorbidity Index, considering it a continuous score and a categorical variable with a cut-off ≥ 4, indicating a 10-year mortality risk ≥ 5% [14]. The composition of the comorbidity variable is described in detail in the Supplementary Material.

We also used a combination of specific age-dependent diseases, disabilities, and functionality to assess individuals’ frailty levels. As previously described in the CIRN SOS Network dataset [15], the total deficit score was the individual’s accumulation of deficits. We converted the deficit score to a frailty index (FI), ranging from 0 to 1 (i.e., FI = (deficit score/n), where n is the number of components extracted from age-dependent diseases, disabilities, and functionality. Frailty was assessed as a three-level categorical variable, according to pre-established cut-offs: non-frail (FI < 0.08), pre-frail (FI ≥ 0.08 and FI < 0.21), and frail (FI ≥ 0.21) [16].

### Cases definitions

A nasopharyngeal (NP) swab was obtained from all patients presenting with symptom onset < = 7 days and an admitting diagnosis compatible with acute respiratory illness, including community-acquired pneumonia, exacerbation of Chronic Obstructive Pulmonary Disease or asthma, unexplained sepsis, any respiratory diagnosis or symptom, or patients with fever (≥ 37.5˚C) presenting with acute coronary syndrome, stroke or any other cardiac diagnosis. Patients with positive NP swabs for influenza were enrolled as cases. For each case, up to two control patients were enrolled who had negative NP swabs. Cases and controls were matched according to age strata (≥ 65y or < 65y) and admission date (within 14 days). Samples from the nasopharynx were used to confirm RSV and influenza infections. WHO-validated real-time RT-PCR was employed to detect influenza A viruses (H1N1 and H3N2 subtypes) and influenza B viruses (Yamagata and Victoria lineages). The Seeplex RV15 One-Step ACE Detection kit was used to identify other respiratory virus aetiologies, including RSV [12, 13].

### Outcomes

Patients were followed from their hospitalization until their hospital discharge or death. The outcomes that were recorded included both the overall and the 30-day mortality, length of hospital stay, and complications (non-invasive ventilation, mechanical ventilation, admission to an Intermediate Care Unit [IMCU] and Intensive Care Unit [ICU]).

### Statistical analysis

Descriptive statistics summarized the sociodemographic and clinical characteristics of the cases. For the survival analysis, deaths after 30 days of hospitalization were right-censored; the Kaplan-Meier analysis estimated the probability of survival over the first 30 days.

A logistic regression model was used to predict 30-day mortality based on age, comorbidity burden, and frailty index. Heteroskedasticity-consistent standard errors (HC1) were used to allow the fitting of the model in the presence of heteroskedastic residuals. All analyses were conducted using was performed in R (version 4.2.2) using RStudio IDE (RStudio 2022.12.0 + 353 “Elsbeth Geranium” release).

## Results

Thirty-three cases of RSV and influenza coinfection were identified in the database. To compare the prevalence of respiratory viruses over the seasons, a total of 8,458 patients were enrolled with data available on multiplex respiratory virus testing. Out of these, 781 patients had missing information on sociodemographic and clinical data and were excluded from the analysis. The remaining 7,677 patients were considered, out of which 3,644 patients were found to be positive for respiratory viruses, with the majority (90%) having Influenza. The selection process for study participants is illustrated in Supplementary Material Figure S1.

Table 1 shows the sociodemographic and clinical characteristics of the cases. The median age was 73 years, with a predominance of females. Most participants over 65 years old were females (66.7%) with a median age of 80.5 years [interquartile range of 68.5 to 87.75]. Most individuals reported living in a private community house and denied a smoking history. The comorbidity burden was relatively low, with 15.2% of the patients presenting a high mortality risk due to their comorbidity (i.e., a CCI score above 4). A detailed description and distribution of comorbid conditions can be found in the supplementary material (Table S1 and Figure S2 and S3). Among those with a CCI score above 4, males were the most common (60%) with a median age of 78 years [interquartile range of 76 to 80]. Frailty status was high, with more than half being considered frail (i.e., FI ≥ 0.21) and requiring regular support for daily living activities. Frail participants were mostly females (64.7%) with a median age of 82 years [interquartile range of 76 to 87]. Influenza A H3N2 was the most common isolated influenza subtype, though there was no information on viral subtype in about one-fourth of the influenza A cases; RSV B composed most of the RSV strains. In addition, most individuals reported receiving the vaccine against influenza in the current or past seasons and received antiviral treatment upon hospitalization.

**Table 1:** Sociodemographic and clinical characteristics of the cases

Parameter	Level	Cases (*N* = 33)
Age	Years, Median [1^st,^ 3^rd^ quartile]	73 [62, 85]
	50–65 years	9 (27.3)
	65–75 years	8 (24.2)
	75–85 years	7 (21.2)
	85 + years	9 (27.3)
Sex, %	Female	22 (66.7)
	Male	11 (33.3)
Baseline living condition, %	Assisted living facility	5 (15.2)
	Community group home	5 (15.2)
	Community private house	22 (66.7)
	Unknown	1 (3.0)
Smoking status, %	Current smoker	8 (24.2)
	Former smoker	6 (18.2)
	Never smoked	19 (57.6)
Charlson Comorbidity Index, CCI	Overall score, Median [1^st,^ 3^rd^ quartile]	1 [1, 2]
	Estimated 10-year mortality risk ≥ 5% (CCI score ≥ 4), %	5 (15.2)
Frailty assessment	Frailty Index, Median [1^st,^ 3^rd^ quartile]	0.22 [0.10, 0.44]
	Non-frail (FI < 0.08), %	6 (18.2)
	Pre-frail (FI ≥ 0.08 & FI < 0.21), %	10 (30.3)
	Frail (FI ≥ 0.21), %	17 (51.5)
Current support, %	Require regular support for activities of daily living	21 (63.6)
	Informal support^a^	19 (57.6)
	Paid formal non-nursing support^a^	13 (39.4)
	Paid formal personal care support^a^	8 (24.2)
	Paid nursing support^a^	6 (18.2)
	The patient or family identifies a need for more support for the patient at this time	11 (33.3)
Influenza Vaccination status, %	Current season vaccination	23 (69.7)
	Never vaccinated	6 (18.2)
	Vaccination in prior seasons only	4 (12.1)
Antiviral prescription, %	Antivirals	26 (78.8)
	No antiviral	7 (21.2)
Influenza season, %	2012/2013 (*N* = 4,438)	10 (30.3)
	2013/2014 (*N* = 5,214)	9 (27.3)
	2014/2015 (*N* = 4,122)	14 (42.4)
Viral strain, %	Influenza	
	Influenza A	29 (87.9)
	H1N1	7 (21.2)
	H3N2	13 (39.4)
	Unavailable subtype	9 (27.3)
	Influenza B	4 (12.1)
	Respiratory Syncytial Virus (RSV)	
	RSV A	12 (36.4)
	RSV B	21 (63.6)

Continuous data are presented as median [1^st^, 3^rd^ quartile] and categorical as absolute (relative) frequencies

^a^Missing information: 12 (36.4%) individuals had no information on informal support, paid formal non-nursing support, paid formal personal care support, or paid nursing support

**Fig. 1:**
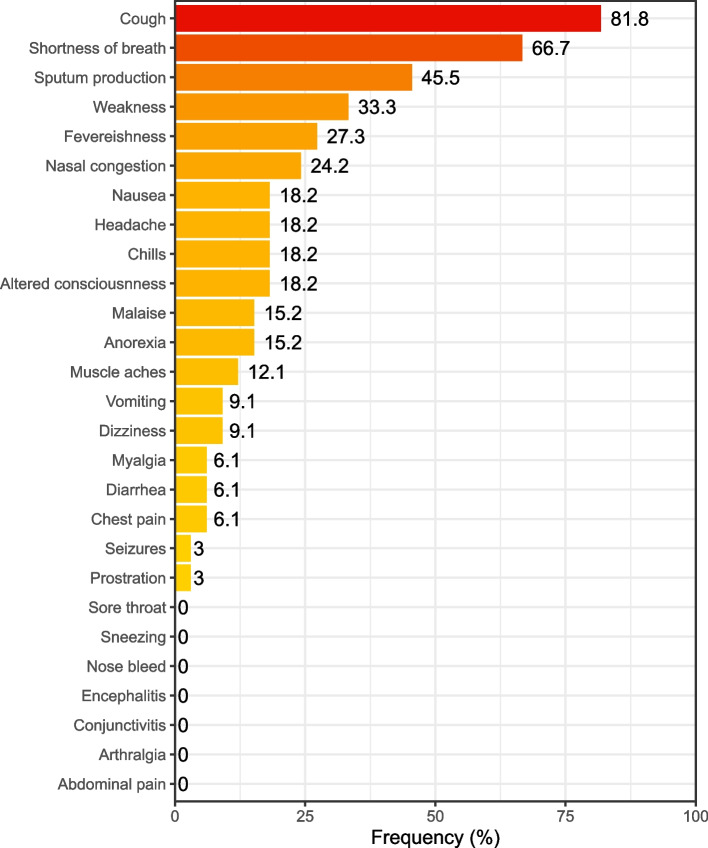
Presenting characteristics of the cases

 Figure 1 demonstrates the presenting characteristics of cases: cough (27/81.8%), shortness of breath (22/66.7%), sputum production (15/45.5%), weakness (11/33.3%), feverishness (9/27.3%) and nasal congestion (8/24.2%) were present in more than 20% of the cases. Altered consciousness, chills, headache, and nausea were reported in 18.2% of cases, followed by anorexia and malaise in 15.2% and muscle aches in 12.1%. Other complaints (vomiting, chest pain, diarrhea, myalgia, prostration, and seizures) were reported between 1 and 10% percentage rate, while abdominal pain, arthralgia, conjunctivitis, encephalitis, nosebleed, sneezing, and sore throat had no reports.

**Fig. 2:**
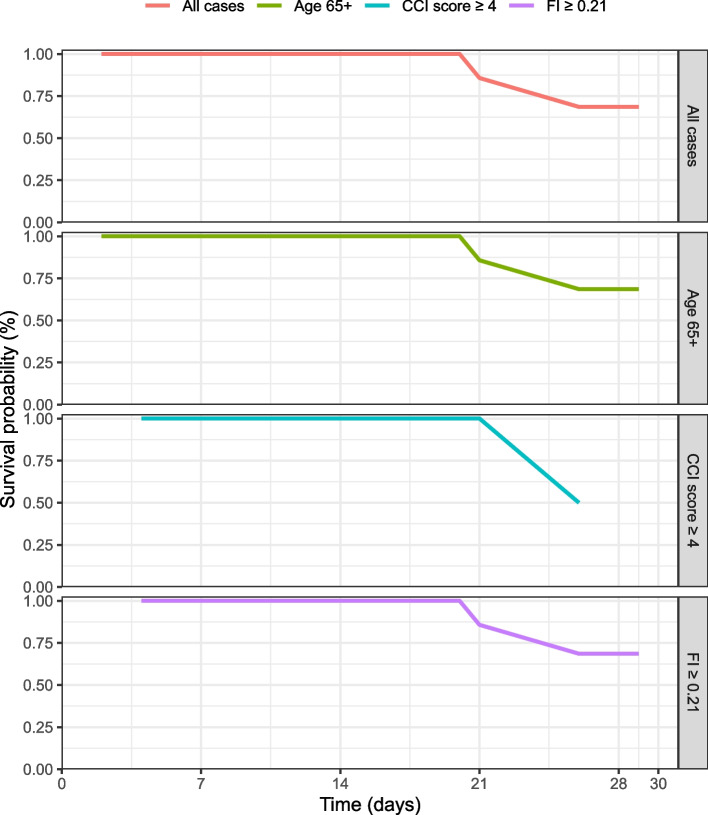
The Kaplan-Meier 30-day survival curve

 Table 2 shows the clinical outcomes observed during the hospital stay. The length of stay varied from 2 to 148 days, with a median of 9 days [1st, 3rd quartile of 4, 20]. The overall mortality was 12.1%, and all the events occurred after the 20th day of hospitalization. The estimated survival probability for deaths occurring before 30 days of hospitalization at day 21 was 85.7% (95% Confidence Interval [CI], 13.2 − 63.3%), and at day 30 was 68.6% (95% CI, 18.6 − 40.3%) (Fig. 2). Of the patients, 27.1% experienced at least one complication during their hospital stay, with the most common being admission to an ICU (21.2%), followed by mechanical ventilation (15.2%) and non-invasive ventilation (9.1%).

**Table 2:** Clinical outcomes observed during the hospital stay

Parameter	All cases (*N* = 33)	Age 65+ (*N* = 24)	CCI score ≥ 4 (*N* = 5)	FI ≥ 0.21 (*N* = 17)
Overall mortality proportion, %	4 (12.1)	4 (16.7)	2 (40.0)	4 (23.5)
30-day mortality proportion, %	2 (6.1)	2 (8.3)	1 (20.0)	2 (11.8)
Length of stay, days	9 [4, 20]	14.5 [8.5, 21]	21 [19, 26]	20 [12, 26]
Complications	9 (27.3)	8 (33.3)	4 (80.0)	7 (41.2)
Non-invasive ventilation (NIV), %	3 (9.1)	3 (12.5)	1 (20.0)	2 (11.8)
Duration of non-invasive ventilation, days	1 [1, 1.5]	1 [1, 1.5]	1 [1]	1.5 [1.2, 1.7]
Mechanical ventilation, %	5 (15.2)	5 (20.8)	3 (60.0)	5 (29.4)
Duration of mechanical ventilation, days	12 [2, 21]	12 [2, 21]	2 [1.5, 11.5]	12 [2, 21]
Admission to Intermediate Care Unit, %	2 (6.1)	1 (4.2)	0 (0.0)	1 (5.9)
Admission to Intensive Care Unit (ICU), %	7 (21.2)	7 (29.2)	4 (80.0)	7 (41.2)
Length of stay in ICU, days	2 [0.5, 8.5]	2 [0.5, 8.5]	8.5 [1.5, 27.5]	2 [0.5, 8.5]
Clinical outcome at discharge				
Died during admission	4 (12.1)	4 (16.7)	2 (40.0)	4 (23.5)
Discharged home	25 (75.8)	16 (66.7)	2 (40.0)	9 (52.9)
Discharged to assisted living	1 (3.0)	1 (4.2)	1 (20.0)	1 (5.9)
Discharged to long-term care	2 (6.1)	2 (8.3)	0 (0.0)	2 (11.8)
Remains in hospital as alternate level of care	1 (3.0)	1 (4.2)	0 (0.0)	1 (5.9)

Continuous data are presented as median [1^st^, 3^rd^ quartile] and categorical as absolute (relative) frequencies

We conducted a logistic regression analysis to predict 30-day mortality based on age, comorbidity burden, and frailty. To prevent any bias that may arise from a particular category having more outcomes, we treated age, CCI score, and FI as continuous variables. The analysis showed that only frailty was significantly associated with 30-day mortality (*p* = 0.03), while age and comorbidity burden were not (*p* = 0.16 and 0.82, respectively).

## Discussion

This report presents the clinical characteristics and outcomes of thirty-three Influenza and RSV coinfection cases registered in the CIRN SOS Network database between 2012 and 2014 out of a total of 3,644 patients who tested positive for a respiratory virus. All coinfection cases occurred in adults aged fifty years and above, with the most common symptoms being constitutional and lower respiratory tract infections. The overall mortality rate of the coinfection cases was significant, at 12.1%, which is higher than most prior reports of either infection alone [12].

Coinfections of the influenza virus with RSV are uncommon but a grave concern in older adults due to the high likelihood of adverse clinical outcomes. Despite this, little is known about coinfection cases’ sociodemographic and clinical characteristics. The first series of influenza virus co-infections with RSV was described in 2020, in which demographic and clinical data from 19 Chinese patients were compared with a control group of patients who were infected with influenza or RSV [5]. The study had several limitations, particularly analytical ones - the authors focused on comparing the characteristics of coinfection cases with those monoinfected in a small sample size. Here, we focused on extensively describing the sociodemographic and clinical characterization of cases of coinfection with influenza and RSV. Our findings agree with a previous report indicating the disease burden among Canadian adults aged 50 and older hospitalized with RSV [12]. This aspect is fundamental to demonstrate that coinfection is indistinguishable from isolated infection with other respiratory viruses regarding symptomatology and to point out the difficulty of establishing a clinical diagnosis for coinfections. This evidence has important implications for clinicians because search-satisfaction bias may stop searching for additional infecting agents after identifying one using sequential testing protocols [17]. This issue will become more relevant as specific therapeutic options become available for each respiratory virus. Moreover, from a sociodemographic perspective, all circumstances occurred in individuals aged 50 or above, requiring special consideration.

The mortality was high in this cohort of patients with coinfection, at 12.1%. This rate is higher than mortality reports in comparable hospitalized populations with one of these viruses at a time. For example, mortality among adults with laboratory-confirmed influenza in the CIRN SOS Network during the 2014/15 season was reported to be 7.9% [18]. Another recent report from our network comparing outcomes from RSV and influenza in the same seasons reported here, 30 days mortality from RSV cases was 6.1% compared to 8.7% for influenza A and 9.1% for influenza B [12]. The cumulative mortality rate over these three years is comparable to the annual mortality rate reported by other studies of RSV mortality in patients with comorbidities [19]. A study based on administrative data in Ontario, Canada, reported death rates among hospitalized patients (all ages) of 7.0% for influenza and 2.9% for RSV [20]. However, data from this paper demonstrated higher mortality when limited to only adults aged 20+ (8.2% for influenza and 10.1% for RSV) [20].

Interpretation of results from active vs. passive/administrative surveillance systems can be complex, given that both viral illnesses likely suffer under-detection due to a lack of universal testing protocols and reliance on clinician recognition of the need to test [21]. Whether this higher mortality is attributable to coinfection vs. older age in those presenting with coinfection (given that mortality increases with age in single and coinfections) is worthy of further investigation.

We found that age over 65 and a high burden of comorbidities (i.e., CCI score > 4) were independently associated with mortality, adverse clinical outcomes, and longer length of stay (both overall and in ICU). Aging is a complex process that affects the functioning and regulation of the immune system: there is an involution of primary lymphoid organs, impairment of phagocytic function, increased levels of pro-inflammatory cytokines, and reduction of naive T and B lymphocytes, accompanied by an increase in dysfunctional lymphocytes [22]. These changes in immune responses, combined with underlying health conditions that increase susceptibility and further impact recovery potential, are linked to greater vulnerability to infections and adverse outcomes. Moreover, older adults, especially those 65 years and older, are at higher risk for severe complications and hospitalization from influenza and RSV mono-infections than younger individuals [23]. Our results agree with these findings, adding to the literature information on predictors of adverse clinical outcomes among older adults with RSV and influenza coinfection.

However, age alone is insufficient to explain the diversity of manifestations and clinical outcomes of individuals of the same age [24]. To address this gap, a broad and holistic approach may help to understand the interaction between various aspects of immune aging and the accumulated effect of changes in health that occur throughout each individual’s life that confer greater vulnerability to infections and adverse outcomes – this is the concept of frailty [25]. Studies have shown that frailty is associated with adverse clinical outcomes in older adults with infectious diseases [26]. Here we found that in cases of influenza and RSV coinfection, frailty was associated with a more severe illness course and a higher risk of complications, hospitalization, and mortality, highlighting the importance of identifying and managing frailty in this population to improve their health outcomes. Frailty was found to be the only significant factor associated with 30-day mortality (*p* = 0.03), while age and comorbidity burden were not significant (*p* = 0.16 and 0.82, respectively) according to logistic regression analysis.

When interpreting the results, one must consider the limitations of this study. The sample size of coinfected patients is relatively small, so generalizations must be made cautiously. The small number of coinfections does not allow for some more detailed assessments of important geriatric outcomes, including changes in frailty and functional status. Additionally, not all sites and seasons of the CIRN SOS Network included multiplex testing for respiratory viruses, potentially leading to bias in epidemiological interpretations. Moreover, the RV15 multiplex testing was used to identify RSV as an endpoint of conventional RT-PCR, so it is likely that some coinfections were missed, as real-time RT-PCR would have been more sensitive. Another limitation is that CIRN SOS surveillance focused on nasopharyngeal swabs, so it is conceivable that some severe cases were missed due to the absence of upper tract swabs that may be negative, even if the lower tract was positive. Despite this limitation, the data set used in this study was extensive: 13,797 patients admitted to the CIRN SOS network with acute respiratory infections were screened, and more than half of the patients (55.4%) underwent multiplex testing.

## Conclusions

Older adults, especially those over 65 with high comorbidity burden and frailty, are at high risk for complications from influenza and RSV coinfections. Clinical diagnosis is unreliable due to similar symptom burdens with single and coinfections. As specific therapeutic and prevention products come into use for individual respiratory viruses, it will be essential to consider comprehensive testing strategies to detect cases of coinfection and improve outcomes, particularly for those most at risk.

### Supplementary Information

**Additional file 1: Figure S1.** Flowchart illustrating the process for selecting study participants. **Table S1.** Absolute and Relative Frequency of Comorbid Conditions in the Charlson Comorbidity Index Among Study Participants. **Figure S2.** Relative Frequency of Comorbid Conditions in the Charlson Comorbidity Index Among Study Participants. **Figure S3.** The frequency at which different values of the Charlson Comorbidity Index appear among the participants in the study.

## Data Availability

The datasets generated and/or analysed during the current study are not publically available due to the confidential nature of the data obtained from patients, however, datasets are available through the corresponding author on reasonable request.
