# Supplementary material for: Clinical features and outcomes of influenza and RSV coinfections: a report from Canadian immunization research network serious outcomes surveillance network
Source: BMC Infect Dis. 2024 Jan 30;24:147. doi: 10.1186/s12879-024-09033-5 (PMC10826021; doi:10.1186/s12879-024-09033-5)

**Supplementary Materials**

This section pertains to the supplementary analysis that was conducted in the study titled " Clinical features and outcomes of influenza and RSV coinfections: A report from Canadian Immunization Research Network Serious Outcomes Surveillance Network".

**Contents**

[**Figure S1. Flowchart illustrating the process for selecting study participants.** 2](#_Toc154028577)

[**Table S1. Absolute and Relative Frequency of Comorbid Conditions in the Charlson Comorbidity Index Among Study Participants.** 3](#_Toc154028578)

[**Figure S2. Relative Frequency of Comorbid Conditions in the Charlson Comorbidity Index Among Study Participants.** 4](#_Toc154028579)

[**Figure S3. The frequency at which different values of the Charlson Comorbidity Index appear among the participants in the study.** 5](#_Toc154028580)

# **Figure S1. Flowchart illustrating the process for selecting study participants.**

**
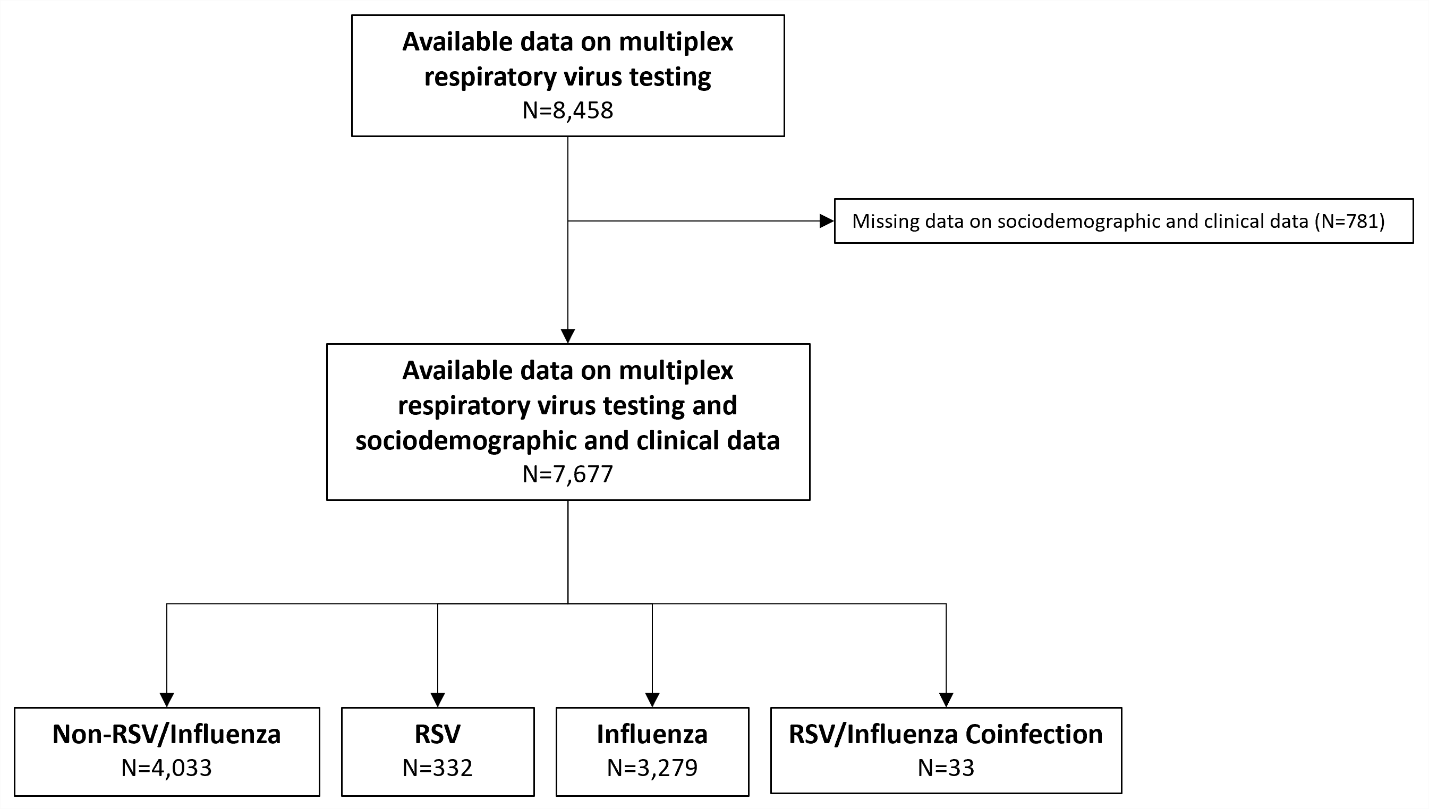
**

# **Table S1. Absolute and Relative Frequency of Comorbid Conditions in the Charlson Comorbidity Index Among Study Participants.**

| **Variable** | **All cases (N=33)** | **Age 65+ (N=24)** | **CCI score ≥ 4 (N=5)** | **FI ≥ 0.21 (N=17)** |
| --- | --- | --- | --- | --- |
| Myocardial infarction | 5 (15.2) | 4 (16.7) | 1 (20.0) | 5 (29.4) |
| Congestive heart failure | 8 (24.2) | 7 (29.2) | 2 (40.0) | 6 (35.3) |
| Peripheral vascular disease | 2 (6.1) | 2 (8.3) | 0 (0.0) | 2 (11.8) |
| Cerebrovascular disease | 8 (24.2) | 6 (25.0) | 5 (100.0) | 8 (47.1) |
| Hemiplegia or paraplegia | 1 (3.0) | 0 (0.0) | 1 (20.0) | 1 (5.9) |
| Dementia | 0 (0) | 0 (0.0) | 0 (0.0) | 0 (0.0) |
| Chronic pulmonary disease | 7 (21.2) | 6 (25.0) | 3 (60.0) | 5 (29.4) |
| Rheumatologic disease | 0 (0) | 0 (0.0) | 0 (0.0) | 0 (0.0) |
| Peptic ulcer disease | 0 (0) | 0 (0.0) | 0 (0.0) | 0 (0.0) |
| Diabetes without chronic complications | 11 (33.3) | 8 (33.3) | 5 (100.0) | 9 (52.9) |
| Diabetes with chronic complications | 2 (6.1) | 1 (4.2) | 2 (40.0) | 2 (11.8) |
| Renal disease | 5 (15.2) | 4 (16.7) | 3 (60.0) | 5 (29.4) |
| Any malignancy, including leukemia and lymphoma | 0 (0) | 0 (0.0) | 0 (0.0) | 0 (0.0) |
| Metastatic solid tumor | 1 (3.0) | 1 (4.2) | 0 (0.0) | 0 (0.0) |
| Mild liver disease | 0 (0) | 0 (0.0) | 0 (0.0) | 0 (0.0) |
| Moderate or severe liver disease | 0 (0) | 0 (0.0) | 0 (0.0) | 0 (0.0) |
| AIDS/HIV | 0 (0) | 0 (0.0) | 0 (0.0) | 0 (0.0) |

Categorical data is presented as absolute (relative) frequencies.

# **Figure S2. Relative Frequency of Comorbid Conditions in the Charlson Comorbidity Index Among Study Participants.**


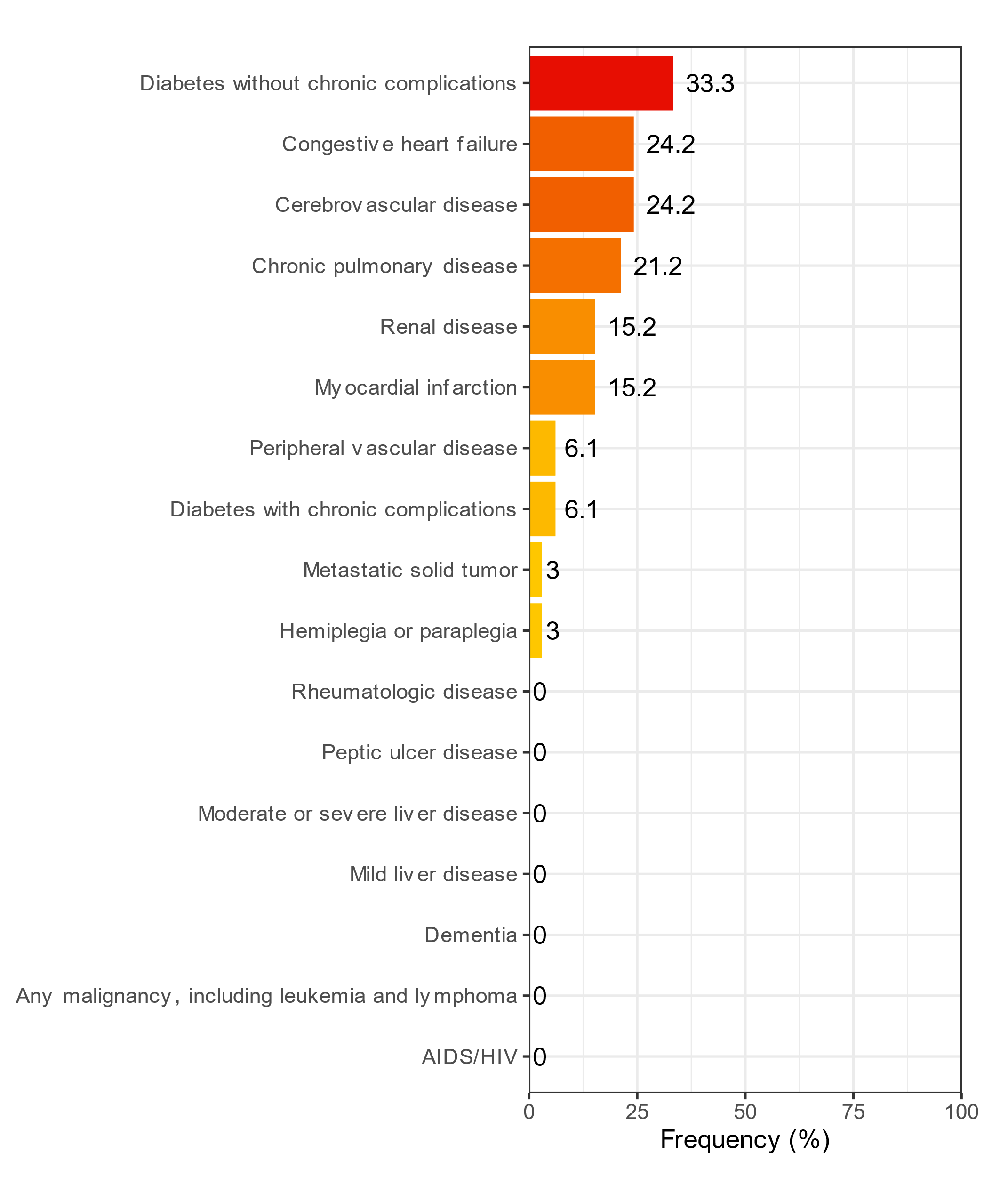


# **Figure S3. The frequency at which different values of the Charlson Comorbidity Index appear among the participants in the study.**


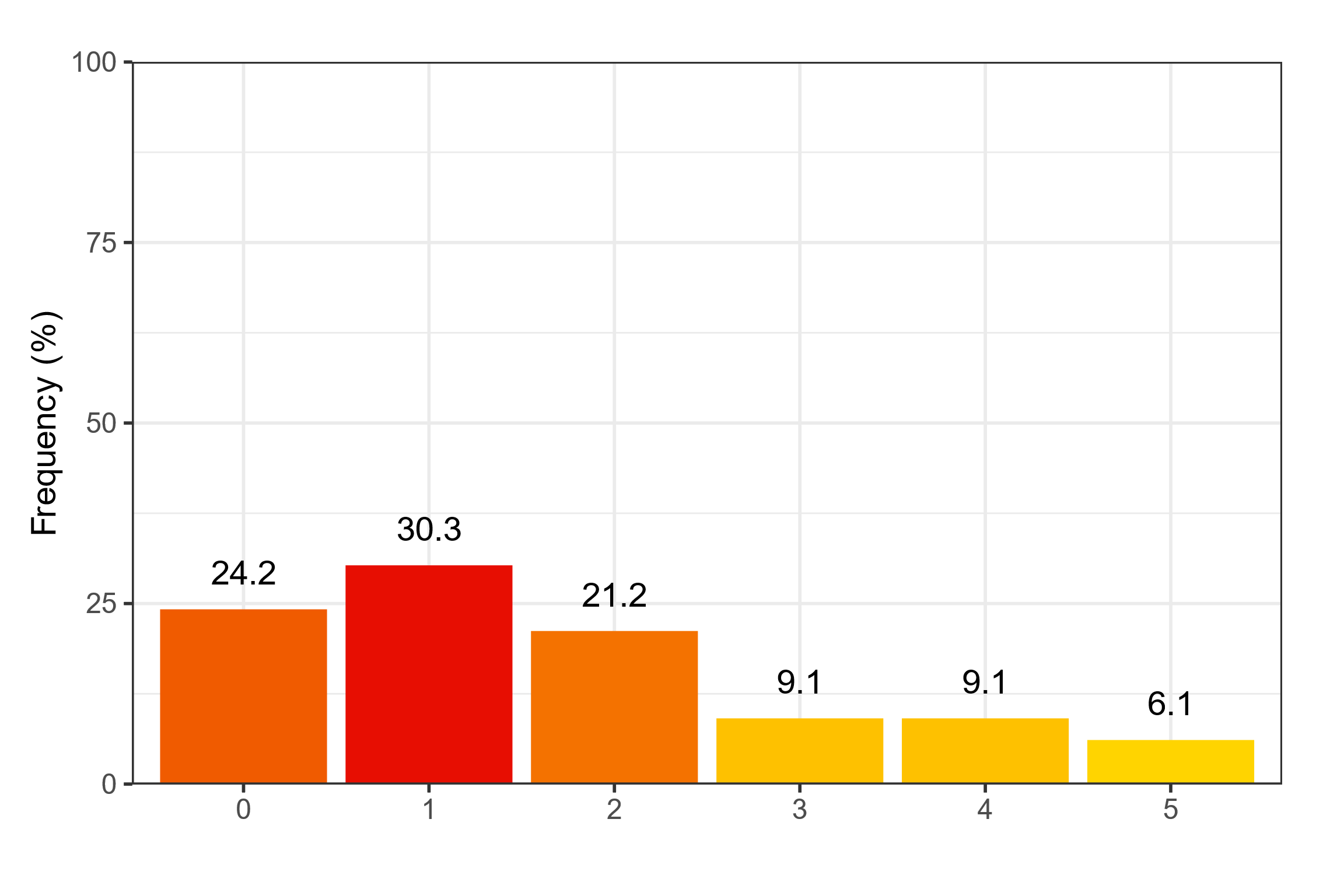

Supplement: Supplementary file 1 — Additional file 1: Figure S1. Flowchart illustrating the process for selecting study participants. Table S1. Absolute and Relative Frequency of Comorbid Conditions in the Charlson Comorbidity Index Among Study Participants. Figure S2. Relative Frequency of Comorbid Conditions in the Charlson Comorbidity Index Among Study Participants. Figure S3. The frequency at which different values of the Charlson Comorbidity Index appear among the participants in the study. [file 12879_2024_9033_MOESM1_ESM.docx]
